# Photochromic Azobenzene Inverse Opal Film toward Dynamic Anti-Fake Pattern

**DOI:** 10.3390/molecules28155881

**Published:** 2023-08-04

**Authors:** Junchao Liu, Zhitong Han, Pingping Wu, Yuanyuan Shang, Jiansheng Chen, Pan Jia

**Affiliations:** 1 Hebei Key Laboratory of Inorganic Nanomaterials, College of Chemistry and Material Science, Hebei Normal University, Shijiazhuang 050024, China; hzt@stu.hebtu.edu.cn (Z.H.); cjs@stu.hebtu.edu.cn (J.C.); 2 School of Sciences, Xi’an University of Technology, Xi’an 710048, China; liujunchao17@mails.ucas.edu.cn; 3 School of Chemistry and Chemical Engineering, Xi′an University of Architecture and Technology, Xi′an 710055, China; pingpingwu@xauat.edu.cn; 4 Hubei Key Laboratory of Processing and Application of Catalytic Materials, College of Chemistry and Chemical Engineering, Huanggang Normal University, Huanggang 438000, China; shangyuanyuan19@mails.ucas.ac.cn

**Keywords:** azobenzene, inverse opal, dynamic fluorescence, photochromism, anti-fake pattern

## Abstract

Azobenzene mesogens have garnered considerable research attention in the realm of photo-responsive materials due to their reversible *trans*–*cis* isomerization. In this paper, we demonstrate an azobenzene inverse opal film synthesized via photo-polymerization from a SiO_2_ opal template. The proposed design exhibits intriguing optical properties, including dynamic fluorescent features, distinct fluorescent enhancement, and an anti-fake micropattern with a switchable structure color. This work holds significant importance for advancing the development of novel optical devices.

## 1. Introduction

Functional fluorescent materials have aroused extensive research interest due to their promising applications in light-emitting devices [1,2], biological imaging [3,4], and catalysis [5,6]. Among these materials, azobenzene mesogens, as a conventional emitting organic material, have attracted attention for their reversible photo-isomerization effect [7,8]. This unique property opens up potential applications in bionic robots [9,10], artificial motors [11,12], artificial muscles [13,14], and energy generators [15,16]. Moreover, the fluorescent properties of azobenzene mesogens can be dynamically regulated by the photo-isomerization process, leading to the development of dynamic fluorescent materials [7,8]. 

Photonic crystals (PCs) with periodic structures possess exceptional light manipulation properties, making them promising candidates for quick sensing [17,18], ultrasensitive detection [19,20], and novel optical devices [21,22,23,24,25]. By introducing PC structures into azobenzene polymers, distinctive applications can be achieved. For example, the reversible bandgap changes in intensity [26] or position [27] have been achieved by the groups of Yu and Kim, respectively, for azobenzene inverse opal films. However, there is a lack of comprehensive research on the functional optical properties of as-prepared azobenzene inverse opal films. The novel properties of azobenzene inverse opal film are expected to be realized based on the reversible photo-isomerization effect of azobenzene mesogens, which is of great importance for the development of functional optical devices. 

Herein, we present an azobenzene inverse opal (AZOIO) film, fabricated by filling a liquid crystal (LC) precursor into a SiO_2_ opal template, followed by photo-polymerization and dissolution of the SiO_2_ opal template. The as-prepared AZOIOs exhibit intriguing optical properties, including dynamic fluorescence, distinct fluorescent enhancements, and an anti-fake micropattern with a switchable structure color. Additionally, AZOIOs demonstrate excellent resistance to friction and high-/low-temperature conditions, rendering them suitable for practical applications in the field of anti-fake patterns. The novel optical properties of the as-prepared AZOIOs, harnessed from the reversible photo-isomerization effect of azobenzene mesogens, hold great significance for the advancement of functional optical devices.

## 2. Results and Discussion

### 2.1. Fabrication Process of AZOIOs

**Figure 1:**
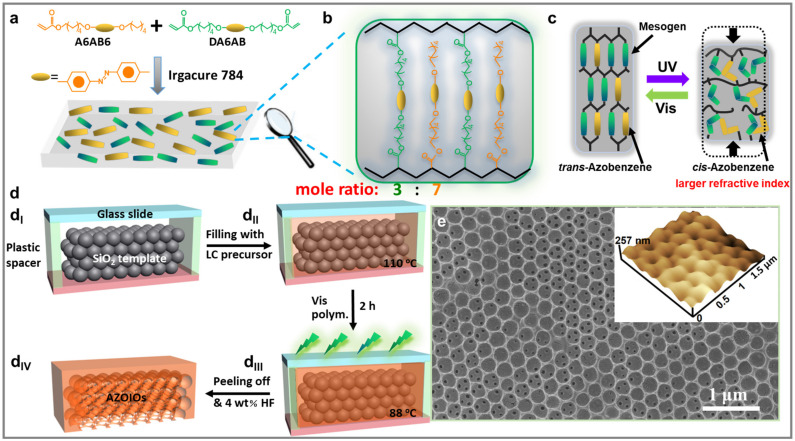
(**a**) Synthesis of bulk azobenzene polymer film and (**b**) corresponding chemical structure. (**c**) Photo-isomerization process for azobenzene mesogens under UV/Vis irradiation. (**d**) Schematic illustration of the fabrication process of AZOIOs. (**d_I_**,**d_II_**) The LC precursor (110 °C) was filled into a unique LC cell consisting of a glass slide and SiO_2_ opal template. (**d_III_**) The system was gradually cooled to 88 °C at a rate of 0.5 °C/min, followed by polymerized under visible light (2 mW/cm^2^, 2 h). (**d_IV_**) A free-standing film was obtained after immersing in HF solution (4 wt%, 24 h). (**e**) Top-view SEM/AFM images of the as-prepared AZOIOs.

Figure 1d and Figure S1 provide a general illustration of the fabrication process of AZOIOs based on photo-polymerization from a SiO_2_ opal template [28,29]. The steps involved in the fabrication process are as follows. Firstly, a glass slide and a SiO_2_ opal template were utilized to create a specialized LC cell (Figure 1(d_I_)). Secondly, the LC precursor, consisting of A6AB6 (6-[4-[2-(4-Hexylphenyl)diazenyl]phenoxy]hexyl 2-propenoate)/DA6AB (4-[(1E)-2-[4-[[6-[(1-oxo-2-propen-1-yl)oxy]hexyl]oxy]phenyl]diazenyl]-,6-[(1-oxo-2-propen-1-yl)oxy]hexyl ester) with 2 mol% Irgacure 784, was filled into the LC cell in its isotropic phase at 110 °C (Figure 1(d_II_)). Thirdly, the system was gradually cooled down to reach the LC phase at 88 °C, with a cooling rate of 0.5 °C/min, followed by visible light polymerization at an intensity of 2 mW/cm^2^ for 2 h (Figure 1(d_III_)). After polymerization, the AZOIOs were obtained by dissolving the SiO_2_ spheres with a 4 wt% HF solution for 24 h (Figure 1(d_IV_)). The polymer network structure and the corresponding photo-isomerization process are depicted in Figure 1a–c. The azobenzene mesogens exhibit a stable *trans* configuration, but upon UV irradiation, the *cis* configuration is achieved. Heating or visible light irradiation, on the other hand, promote the conversion of the *cis* to *trans* configuration due to the thermodynamic instability of the *cis* configuration [30]. The isomerization of azobenzene mesogens leads to the reversible effective refractive index transformation under alternating UV/Vis irradiation (Figure 1c) [31].

Observations from scanning electron microscopy (SEM)/atomic force microscopy (AFM) images in Figure 1e and Figure S2 show that the as-prepared AZOIOs display a periodic pore structure of approximately 230 nm. The inner pore structure is also clearly visible from the top view. Furthermore, AZOIOs demonstrate thermostability (Figure S3) and polydomain characteristics (Figure S4).

### 2.2. The Modulation Effect of AZOIOs’ Bandgap and Photo-Isomerization on Fluorescent Properties

**Figure 2:**
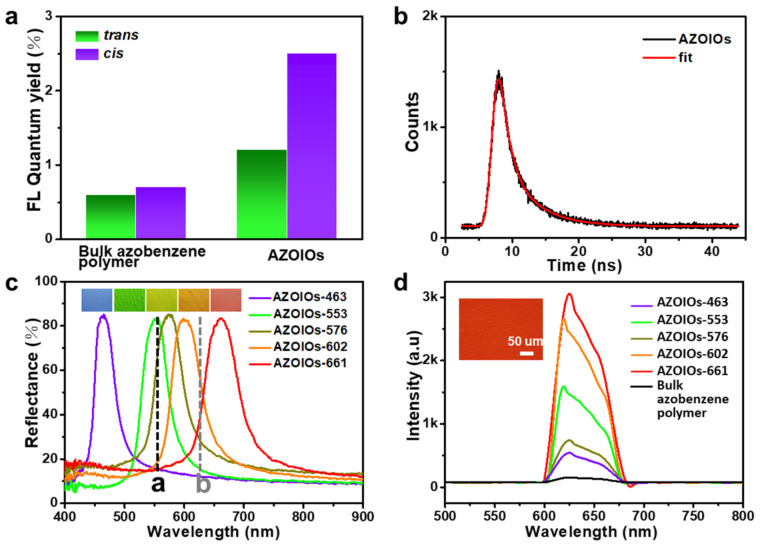
(**a**) Photoluminescence absolute quantum yields of bulk azobenzene polymer and AZOIOs in the *trans* or *cis* state when excited at 560 nm. (**b**) Luminescence decay of AZOIOs when excited by a 560 nm laser. (**c**) Reflectance spectra of AZOIOs with different bandgaps. The peaks of the excitation light (black dotted Line a) and the maximum emission peak of the bulk azobenzene polymer (gray dotted Line b) were located at 560 nm and 625 nm, respectively. The inserts show the corresponding optical images of AZOIOs. (**d**) Emission spectra of AZOIOs with different bandgaps and the bulk azobenzene polymer when excited at 560 nm. The insert displays the corresponding fluorescent image of the AZOIOs.

The photoluminescence absolute quantum yields of AZOIOs in the *trans*/*cis* state when excited by 560 nm were 0.6/0.7% (bulk azobenzene polymer) and 1.2/2.5% (AZOIOs), respectively (Figure 2a). These data preliminarily indicated that the photo-isomerization of azobenzene mesogens and the periodic PC structure had a significant impact on the fluorescent properties of the samples. Additionally, the luminescence decay of AZOIOs when excited by 560 nm was 2.95 ns (Figure 2b and Figure S7). Interestingly, the as-prepared AZOIOs demonstrated controllable fluorescent intensity based on the modulation effect of the bandgap and photo-isomerization.

The maximum emission peak of the AZOIOs (gray dotted Line b) was located at 625 nm when excited by a 560 nm laser (black dotted Line a) (Figure 2c). The AZOIOs displayed a unique fluorescent enhancement (Figure 2d), and a significant influence of the stopband on the fluorescent intensity of the AZOIOs was observed. This influence was mainly due to the relationship between the emission and excitation spectra and the position of the stopband [32,33,34]. The different AZOIO samples are denoted as follows: AZOIO_ex_, with a bandgap overlapping the excitation spectra of the laser; AZOIO_em-red_/AZOIO_em-blue_, with a red/blue band edge of the stopband overlapping the emission spectra of the bulk azobenzene polymer; AZOIO_no_, with a bandgap overlapping neither the excitation spectra of the laser nor the emission spectra of the bulk azobenzene polymer. 

Figure 2c illustrates the reflectance spectra of the AZOIOs (solid line), the excitation light peak (black dotted Line a), and the emission peak (gray dotted Line b) of the bulk azobenzene polymer. The samples of AZOIO-661, -602, -576, -553, and -463 represent AZOIOs with stopbands of 661, 602, 576, 553, and 463 nm, respectively. Figure 2d shows the fluorescent emission spectra of the bulk azobenzene polymer and the AZOIOs when excited by 560 nm laser. Compared to the bulk azobenzene polymer, AZOIO_em-blue_ (AZOIO-661) achieved the most-significant fluorescent enhancement of 19.78-fold, while AZOIO_em-red_ (AZOIO-602) had a 17.11-fold fluorescent enhancement. Similarly, AZOIO_ex_ (AZOIO-553) displayed a 10.28-fold enhancement, and AZOIO_no_ (AZOIO-576 and AZOIO-463) exhibited 4.78-fold and 3.94-fold fluorescent enhancements, respectively. The sample demonstrated a bright red fluorescent color when excited by a 560 nm laser (Figure 2d and Figure S6). A positive fluorescent enhancement was observed for all AZOIOs with different bandgaps, resulting from the periodic arrangement of the PC efficiently exciting the fluorescence due to the high-intensity near-field generated by the interaction with the excitation/emission laser [32,33,34]. 

Notably, the fluorescent intensity of the sample could be effectively controlled by the reversible photo-isomerization process of the azobenzene mesogens. Generally, no fluorescence was observed in the azobenzene solution at room temperature. However, the fluorescence phenomenon of the azobenzene compound can be induced by the bimolecular aggregation of azobenzene chromophore through changes in temperature, solvents’ effects, and light irradiation. The aggregated azobenzene with long alkyl chain increases with the prolongation of UV irradiation time, resulting in a correspondingly enhanced fluorescent intensity. That is, the transformation from the *trans*- to *cis*-azobenzene mesogen contributes to a stronger fluorescent intensity [7,8]. 

The change in fluorescent intensity of *cis*-bulk azobenzene polymer (a fresh bulk azobenzene polymer irradiated by UV light at 30 mW/cm^2^ for 1 min) under visible light irradiation (10 mW/cm^2^) for different times was investigated (Figure S8)**.** The fluorescent intensity of *cis*-bulk azobenzene polymer decreased from 1015 to 940, 900, 870, 855, 840, 826, and 817 a.u. with a UV irradiation time of 3, 6, 10, 15, 20, 25, and 30 min, respectively, corresponding to a decreasing rate of 25, 13.3, 7.5, 3, 3, 2.8, and 1.8 a.u./min. Notably, the decreasing rate in the first half stage (0–10 min) was higher than that in the latter half stage (15–30 min). This phenomenon can be explained by the combined contribution of the photo-isomerization process and photobleaching to the decreased fluorescent intensity in the first half stage, while the change in fluorescent intensity in the latter half stage was mainly induced by photobleaching due to the fast photo-isomerization process of the azobenzene mesogens.

**Figure 3:**
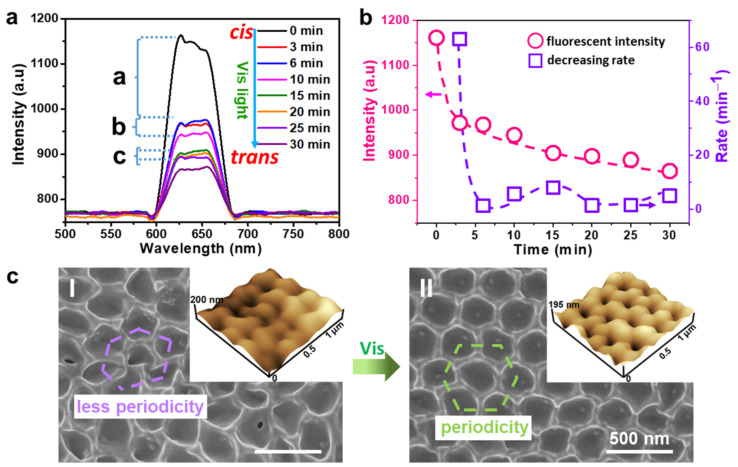
(**a**) Fluorescent intensity changes for *cis*-AZOIOs (fresh AZOIOs irradiated by UV light at 30 mW/cm^2^ for 1 min) under visible light irradiation (10 mW/cm^2^) for different time. (**b**) Transformation of fluorescent intensity and fluorescent decreasing rate for *cis*-AZOIOs as a function of visible irradiation time. SEM and AFM images of *cis*-AZOIOs (**c**-**I**) before and (**c**-**II**) after visible light irradiation.

In comparison, the change in fluorescent intensity for *cis*-AZOIOs (fresh *trans*-AZOIOs irradiated by UV light at 30 mW/cm^2^ for 1 min) under visible irradiation (10 mW/cm^2^) for different durations was investigated (Figure 3a,b). The fluorescent intensity of *cis*-AZOIOs gradually decreased with increasing UV irradiation time. Specifically, the fluorescent intensity of *cis*-AZOIOs transformed from 1161 to 972, 968, 945, 905, 898, 890, and 865 a.u. for a UV irradiation time of 3, 6, 10, 15, 20, 25, and 30 min, respectively, with corresponding decreasing rates of 63, 1.33, 5.75, 8, 1.4, 1.6, and 5 a.u./min. Notably, the entire process could be divided into three stages: a peak rapidly descending period (a) and two plateau periods (b and c). The peak rapidly descending period (a) was caused by the fast photo-isomerization process of azobenzene mesogens and photobleaching, while the appearance of the plateau period (b and c) could be attributed to the enhancement effect of the PC structure on the fluorescent intensity based on the recovery of the PC structure, which slowed down the decreasing rate in the fluorescent intensity to some extent. In detail, the photo-isomerization process from the *trans*-rod-like azobenzene mesogens to a *cis*-bent shape caused a contraction of the sample’s surface. The direction of contraction was not uniform on the sample’s surface due to the polydomain property of AZOIOs (Figure S4), leading to the disturbance of the initial regular periodic pore structure (Figure 3(c-I)). Subsequently, the *cis*-bent azobenzene mesogens transformed back to the *trans*-rod-like shape under visible light irradiation, contributing to a partial recovery of the periodic structure (Figure 3(c-II)). In summary, the periodic pores of the AZOIOs underwent a dynamic change from regular to irregular or irregular to regular in response to UV or visible light [31]. This dynamic fluorescent material allows for easy adjustment of the fluorescent properties according to specific needs.

### 2.3. Applications in Anti-Fake Patterns

**Figure 4:**
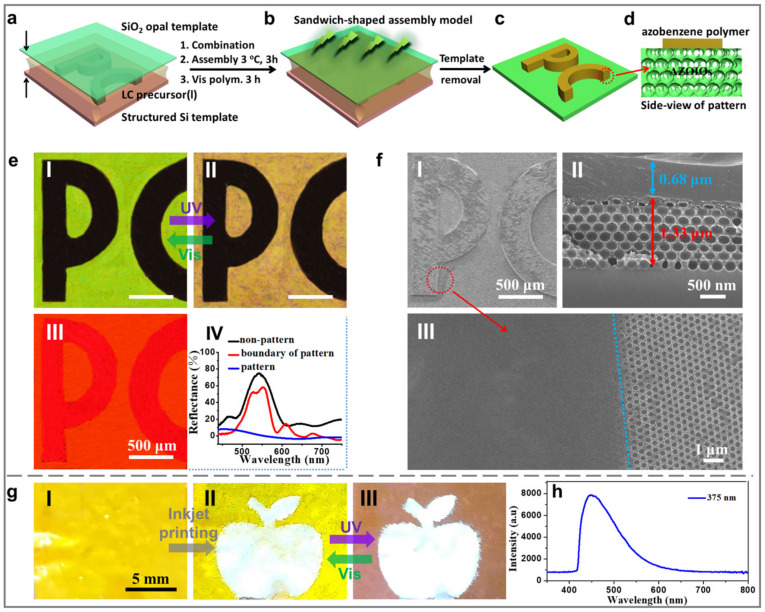
Fabrication process of bilayer patterned AZOIOs. (**a**,**b**) A structured Si template with the letter “PC” was placed horizontally. The LC precursor solution (a mixture of dichloromethane and ethanol in a volume ratio of 1:1) was carefully dropped into the structured Si template, and then, a SiO_2_ opal template was applied on top (3 °C, 3 h). Subsequently, the system was polymerized under visible light (3 mW/cm^2^, 3 h). (**c**) The precisely assembled patterned AZOIOs could be obtained by removing the Si template and SiO_2_ opal template. (**d**) Side-view of patterned AZOIOs. (**e**) Optical images of patterned AZOIOs showing (**I**) before and (**II**) after UV irradiation. (**III**) Fluorescent image of patterned AZOIOs when excited at 560 nm. (**IV**) Reflectance spectra of different regions of the pattern. SEM images of the as-prepared pattern: (**f**-**I**) top view; (**f**-**II**) side view; (**f**-**III**) boundary area. (**g**) Patterned AZOIOs constructed by inkjet printing and the corresponding pattern under UV irradiation. (**h**) Emission spectra of carbon dots when excited at 375 nm.

Figure 4 presents a facile fabrication strategy based on a structured Si template to construct bilayer PC patterns [35,36,37]. The bilayer PC pattern was achieved by sandwiching an LC precursor solution (a mixture of dichloromethane and ethanol in a volume ratio of 1:1) containing A6AB6, DA6AB, and Irgacure 784 between the hydrophilic SiO_2_ opal template and the heptadecafluorodecyltrimethoxysilane (FAS)-treated superhydrophobic structured Si template (Figure 4a and Figure S10). The system was then cooled to 3 °C for 3 h to allow the limited area assembly on the raised “PC” pattern (Figure 4b). A micrometer-scale liquid film was observed between the Si template and the top surface of the letter “PC” structure, creating by the wetting defects of the raised “PC” structure. This liquid film provided a space for the effective aggregation of the LC precursor solution [35,36,37]. Afterward, the assembly system was polymerized under visible light (3 mW/cm^2^, 3 h; Figure 4b). Precisely assembled patterned AZOIOs could be obtained by removing the Si template and SiO_2_ opal template (Figure 4c). The final pattern displayed a black “PC” pattern of redundant bulk azobenzene polymer (Figure 4(f-II)) written on the surface of the green AZOIO background (Figure 4(e-I)).

As shown in the SEM image (Figure 3(f-III)), a typical inverse opal structure was observed on the right side, while the pores on the left side were covered by an extremely thin bulk azobenzene polymer. The selective aggregation of the bulk azobenzene polymer on the surface of the AZOIOs contributed to the formation of the “PC” pattern. The thickness of the bulk azobenzene polymer (upper layer) was approximately 0.68 μm, while the inverse opal structure (bottom layer) was approximately 1.33 μm (Figure 4(f-II)). The pattern presented a bright red fluorescent color when excited by a 560 nm laser (Figure 4(e-III)). Notably, the “PC” pattern area exhibited a stronger fluorescent intensity compared to the background region due to the unique bilayer structure (Figure 4d,(f-II)). Specifically, the upper layer of the bulk azobenzene polymer displayed further fluorescent enhancement based on the existence of a dielectric mirror (inverse opal structure) [38,39], which allowed more laser to be located in the bulk azobenzene polymer layer. 

The as-prepared AZOIO pattern presented dynamic color transition properties based on the reversible effective refractive index change [31]. Specifically, the structure color of the pattern’s background transformed from its original green (Figure 4(e-I)) to brown-yellow upon being irradiated by UV light (120 mW/cm^2^) for 2 s (Figure 4(e-II) and Figure S11). This phenomenon could be attributed to the increase in the effective refractive index of the azobenzene mesogens after UV irradiation. Furthermore, Bragg’s law was used to calculate the increased refractive index of the azobenzene mesogens after UV irradiation: *m* × *λ_max_ = 2d* × *n_avg_* × sin *θ*, where *λ_max_* is the maximum wavelength of the reflectance peak (i.e., the stopband position), *m* is the order of the Bragg diffraction (i.e., *m* = 1), *d* is the interplanar spacing between (hkl) planes (i.e., the (111) planes), and *n_avg_* is the average refractive index of the photonic structure. In this work, all reflectance spectra were measured at near-normal incidence to the (111) planes, *θ* = 90°, sin *θ =* 1. The average refractive index of the AZOIOs increased from 1.196 to 1.329 with an increasing degree of 11.1% after UV irradiation. Additionally, the increased degree of the pore size was approximately 4% (Figure S12). The side-view SEM images of the AZOIOs show that the pore structure in the bottom area remained unchanged since only the surface region (∼1 μm) of the film contracted due to the strong absorption coefficient of the azobenzene mesogens toward UV light (Figure S13). The change in the refractive index of azobenzene mesogens after UV irradiation was the leading factor for the structure color change in the sample. 

Subsequently, the structure color of the pattern’s background recovered to its initial green state after being irradiated by visible light (120 mW/cm^2^, 2 s) due to the decreased effective refractive index of the azobenzene mesogens (Figure 4(e-I)). No obvious phase transition was observed under UV irradiation (120 mW/cm^2^, 2 s; Figure S5a) due to the highest temperature of the sample (*ca.* 34.8 °C) being much lower than the phase transition temperature (*ca*. 150 °C; Figure S3b). There was no significant difference observed between the polarized optical microscopy (POM) images of the sample before and after UV irradiation (120 mW/cm^2^, 2 s; Figure S5b). The color transformation of the as-prepared bilayer AZOIO pattern endowed it with a dynamically changing performance, making it suitable for the fabrication of anti-fake patterns. Furthermore, the reflectance spectra of different regions of the pattern were measured (Figure 4(e-IV)). The boundary of the pattern area had a broad dip in reflectance due to the presence of the thin bulk azobenzene polymer, which could be attributed to the excitation of photonic resonances at crystal boundaries [39].

In contrast, the pattern area demonstrated no reflectance signal, caused by the thick bulk azobenzene polymer layer. Additionally, inkjet printing was utilized to construct patterned AZOIOs (Figure 4g) [40,41], where carbon dots were used as the ink due to its strong fluorescence property (Figure 4h) [42,43]. Similarly, the as-prepared pattern demonstrated dynamic color change under alternating UV/Vis irradiation (Figure 4(g-II,III)). The combination of the dynamic structure color and complex microstructure makes the as-prepared pattern applicable in the field of anti-fake technology. 

### 2.4. Stability Test of AZOIOs

**Figure 5:**
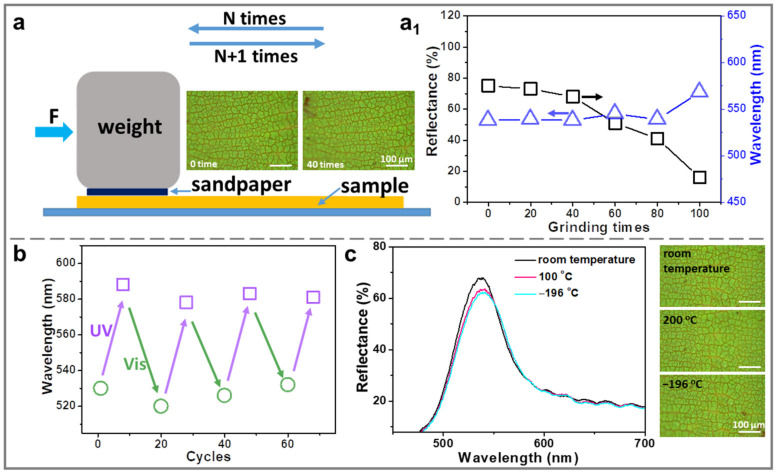
(**a**) Diagram of the sandpaper abrasion test setup. A weight of 10 g was applied to a sandpaper piece with a size of 0.9 × 0.8 cm. (**a_1_**) Changes in the reflection peak and reflectance of the AZOIOs after different abrasion times. (**b**) Variation of the AZOIOs’ bandgap during 70 cycles under alternating UV/Vis irradiation. (**c**) Reflectance spectra of the AZOIOs at different temperatures and corresponding optical images.

To ensure practical applications, the stability of the AZOIOs was evaluated in terms of abrasion resistance, switchable color cycle, and low-temperature stability (Figure 5). For the abrasion resistance test, a piece of grit sandpaper (0.9 × 0.8 cm) was attached to a weight (10 g). The pressure applied on the abrasion area (S) was calculated to be approximately 1389 Pa using P = F/S = mg/S. During the test, the sandpaper, with the weights on it, was placed on the surface of the sample and moved manually in a parallel direction. After every 10 cycles, the sandpaper was blown with N_2_ to remove the rubbed-off material. The coating surfaces were also blown with N_2_ after the abrasion (Figure 5a) [44]. As a result, the AZOIOs exhibited robust abrasion resistance. The reflectance peak of the AZOIOs remained almost unchanged in the first 40 abrasion cycles, with only a slight decrease in reflectance intensity (Figure 5(a_1_)). From 40 to 100 abrasion cycles, wear marks appeared on the surface of the AZOIOs (Figure S14). Additionally, the switchable color of the AZOIOs under alternating UV/Vis irradiation could be repeatedly cycled for more than 70 cycles without any significant delay (Figure 5b). Finally, the resultant AZOIOs demonstrated high-/low-temperature resistance. For example, the reflectance peak showed little change in intensity and position when the sample was exposed to 100 °C or −196 °C for 1 min, compared to room temperature (Figure 5c). This indicates that the AZOIOs can maintain their optical properties even under extreme temperature conditions.

### 2.5. Conclusions

In conclusion, a variable fluorescent material was achieved by incorporating fluorescent azobenzene mesogens into a PC structure. The as-prepared sample demonstrated variable fluorescent properties based on the PC structure. The switchable structure color, resulting from the periodic PC structure, could be easily controlled under alternating UV/Vis irradiation due to the reversible change of the sample’s effective refractive index. Moreover, the dynamic anti-fake pattern showcased the potential for practical applications. The findings of this research significantly broaden the applications of azobenzene materials in novel optical devices. 

## 3. Materials and Methods

### 3.1. Self-Assembly of SiO_2_ Opal Template

The SiO_2_ opal template was prepared by vertically depositing a clean glass substrate into a vial containing SiO_2_ colloidal suspensions (0.15 wt.%) at 60 °C with a relative humidity of 60% for 48 h. 

### 3.2. Fabrication of Azobenzene Monomer/Crosslinker

A6AB6 and DA6AB were synthesized following previously reported procedures [28,29]. 

### 3.3. Fabrication of Azobenzene Inverse Opals

The AZOIOs were fabricated as follows: A mixture of 20 mg A6AB6, 10 mg DA6AB, and chloroform solvent of Irgacure 784 (1.0 mg/mL, 0.71 mL) was evaporated in the dark state. The resulting mixture was melted at 110 °C (isotropic phase) and injected into an LC cell composed of the SiO_2_ opal template and a clean glass slide. The sample was slowly cooled down (0.5 °C/min) to the polymerization temperature (88 °C, LC phase). Then, photo-irradiation was conducted at >540 nm (2 mW/cm^2^) with a high-pressure mercury lamp through glass filters for 2 h. Finally, the composite film was peeled off after polymerization and immersed in HF (4 wt.%) for 24 h to dissolve the SiO_2_ opal template. 

### 3.4. Groove-Structured Si Templates

The Si wafers with the template (N-doped, <100>-oriented, 400 μm thick, 1 cm diameter) were structured using a direct laser writing apparatus (DWL200, Heidelberg Instruments Mikrotechnik, Heidelberg, Germany) to transfer the computer-predefined design onto the photoresist-coated (Shipley Microposit S1800 series, Shipley Co., Ltd., Tokyo, Japan) wafer with about 1 μm precision. After irradiation and development, the wafers were etched using deep reactive ion etching (DRIE}) (Alcatel 601E) with fluorine-based reagents for different times (10 s to 6 min) depending on the desired height of the structures. Groove-structured Si templates with a “PC” pattern were fabricated. After resist stripping (Shipley Microposit Remover 1165, Shipley Co., Ltd., Tokyo, Japan), the templates were cleaned with ethanol and acetone prior to use.

### 3.5. Manufacturing of Anti-Fake Pattern by Structured Si Template

First, the structured Si template (1 cm × 1 cm) was treated by heptadecafluorodecyltrimethoxysilane (FAS) for 6 h to ensure superhydrophobicity (150° water contact angle (CA)), while the SiO_2_ opal template was treated by plasma for 60 s to reduce the water CA on its surface to less than 10°. Secondly, the pattern was created by sandwiching the LC precursor (a mixed solvent of ethanol and dichloromethane with an equal volume ratio) between the superhydrophobic structured Si template and the hydrophilic SiO_2_ opal template, forming a fixed-gap sandwich assembly system. Thirdly, the system was kept at 3 °C for 3 h. Subsequently, the assembly system was polymerized using a high-pressure mercury lamp (>540 nm, 3 mW/cm^2^) for 3 h. After removing the Si template by physical peeling and dissolving the SiO_2_ opal template by HF (4 wt%, 24 h), precisely assembled patterned AZOIOs could be generated. 

### 3.6. Characterization

Optical and polarized optical microscopy (POM) images were obtained using OLYMPUS BX53. The reflectance spectra were recorded using an Ocean Optics Maya 2000 PRO fiber optical spectrometer. The UV light and visible light were obtained from an H086-425 and an HLV2-22RD-3W, respectively. The SEM images were taken with a Thermo-4800 high-resolution field emission scanning electron microscope. Accurate temperature control was achieved using a hot stage (HCS402). Thermodynamic analysis was carried out on a differential scanning calorimetry (DSC6200) at a heating/cooling rate of 10 °C min^−1^ with N_2_ as the protecting atmosphere. Thermogravimetric analysis (TGA) was conducted on Q5000IR from 25 to 500 °C at a heating rate of 10 °C min^−1^ with N_2_ as the protecting atmosphere. The oxygen plasma instrument (DT-03) was from Suzhou OPS oxygen plasma Technology Co., Ltd. Fluorescent emission spectra were recorded on an F-4500. 

## Data Availability

The data presented in this study are available in the Supplementary Materials.

## Supplementary Materials

The following are available online at https://www.mdpi.com/article/10.3390/molecules28155881/s1, Figure S1: SEM and corresponding optical images of the fabrication process for the AZOIOs; Figure S2: SEM images of the as-prepared AZOIOs; Figure S3: TGA and DSC curves of the AZOIOs; Figure S4: POM images of the AZOIOs; Figure S5: The temperature change and POM images of the AZOIOs; Figure S6: Fluorescent images of the glass, the boundary of the AZOIOs, and the AZOIOs; Figure S7: The luminescence decay of the AZOIOs that were excited by 560 nm; Figure S8: The change of fluorescent intensity for the bulk azobenzene polymer under alternating UV/Vis irradiation; Figure S9: AFM images of the AZOIOs under alternating UV/Vis irradiation and the corresponding Fourier transform. Figure S10: SEM images of the patterned bulk azobenzene polymer on a cover glass; Figure S11: Reversible bandgap shift of the AZOIOs under alternating UV (365 ± 20 nm, 120 mW/cm^2^)/Vis (560 ± 20 nm, 120 mW/cm^2^) irradiation; Figure S12: SEM images of the AZOIOs after UV irradiation (20 mW/cm^2^) with different times. Figure S13: Side-view SEM images of the AZOIOs after UV irradiation (20 mW/cm^2^, 20 s). Figure S14: In situ observation of reflection spectra and corresponding optical images of the AZOIOs after different abrasion times.

## Sample Availability

Samples of the compounds, films, and patterns are available from the authors.
