# Supplementary material for: Photochromic Azobenzene Inverse Opal Film toward Dynamic Anti-Fake Pattern
Source: Molecules. 2023 Aug 4;28(15):5881. doi: 10.3390/molecules28155881 (PMC10421165; doi:10.3390/molecules28155881)
Supplement: Supplementary file 1 [file molecules-28-05881-s001.zip › molecules-2502125-supplementary.pdf]

Supporting Information

Photochromic Azobenzene Inverse Opal Film toward Dynamic Anti-Fake Pattern

Junchao Liu [2], Zhitong Han [1], Pingping Wu [3], Yuanyuan Shang [4], Jiansheng Chen [1] and Pan Jia [1,*]

[1] Hebei Key Laboratory of Inorganic Nanomaterials, College of Chemistry and Material Science,
Hebei Normal University, Shijiazhuang 050024, China; hzt@stu.hebtu.edu.cn (Z.H.);
cjs@stu.hebtu.edu.cn (J.C.)

[2] School of Sciences, Xi'an University of Technology, Xi'an 710048, China; liujunchao17@mails.ucas.edu.cn

[3] School of Chemistry and Chemical Engineering, Xi'an University of Architecture and Technology,
Xi'an 710055, China; pingpingwu@xauat.edu.cn

[4] Hubei Key Laboratory of Processing and Application of Catalytic Materials, College of Chemistry and Chemical Engineering, Huanggang Normal University, Huanggang 438000, China; shangyuanyuan19@mails.ucas.ac.cn

* Correspondence: jiapan@hebtu.edu.cn

Table of Content:

1. Fabrication process of AZOIO.

2. The modulation effect of AZOIOs' bandgap and photo-isomerization on fluorescent property.

1. Fabrication process of AZOIO

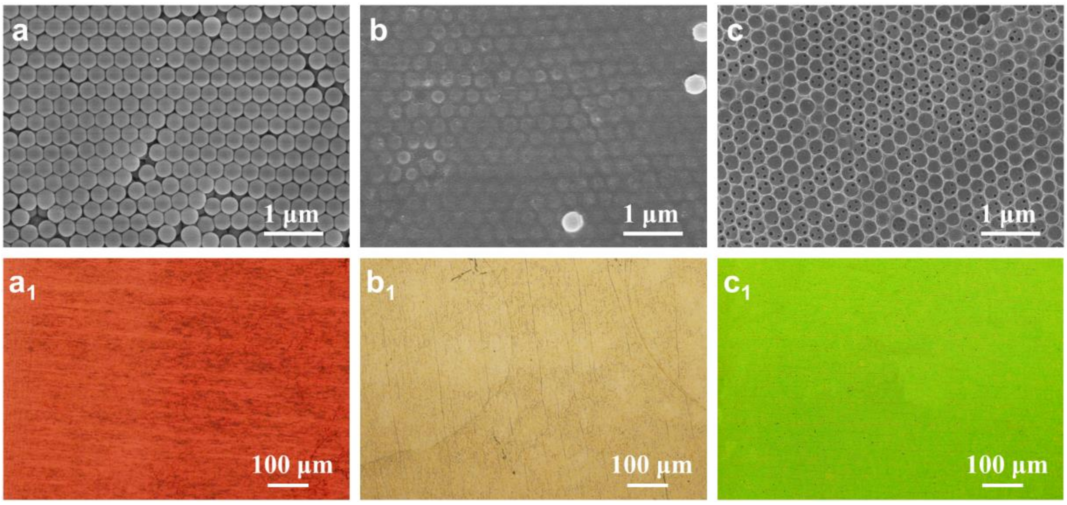

Figure S1. SEM and corresponding optical images of the fabrication process for AZOIOs. (a, a_1) SiO_2 opal template, (b, b_1) composite opal (the interstice of SiO_2 opal template was infiltrated by LC precursor), and (c, c_1) AZOIOs.

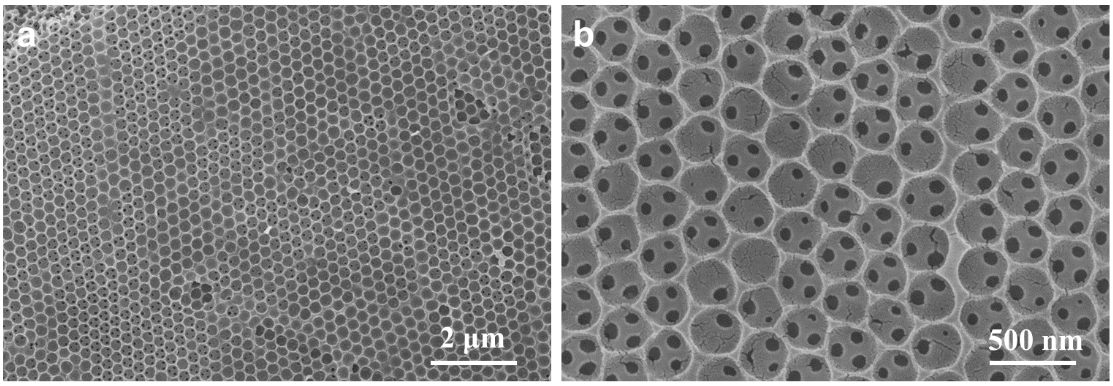

Figure S2. SEM images of the as-prepared AZOIOs.

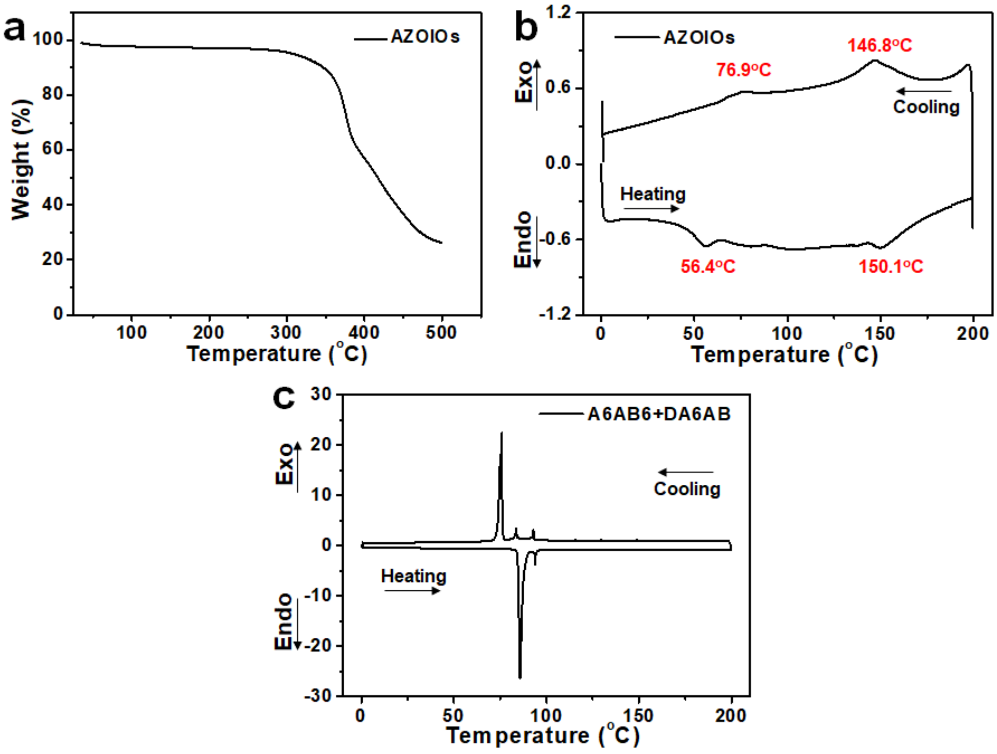

Figure S3. (a) TGA of AZOIOs. (b) DSC curves from the second scan (±10 °C/min) of AZOIOs. (c) DSC curves from the second scan (±10 °C/min) of the monomeric mixture.

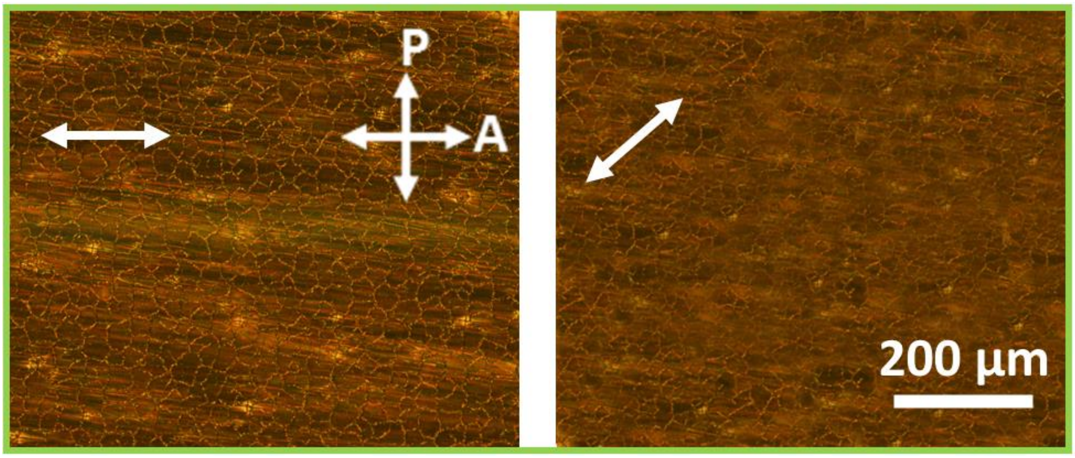

Figure S4. POM images of AZOIOs. P: polarizer, A: analyzer.

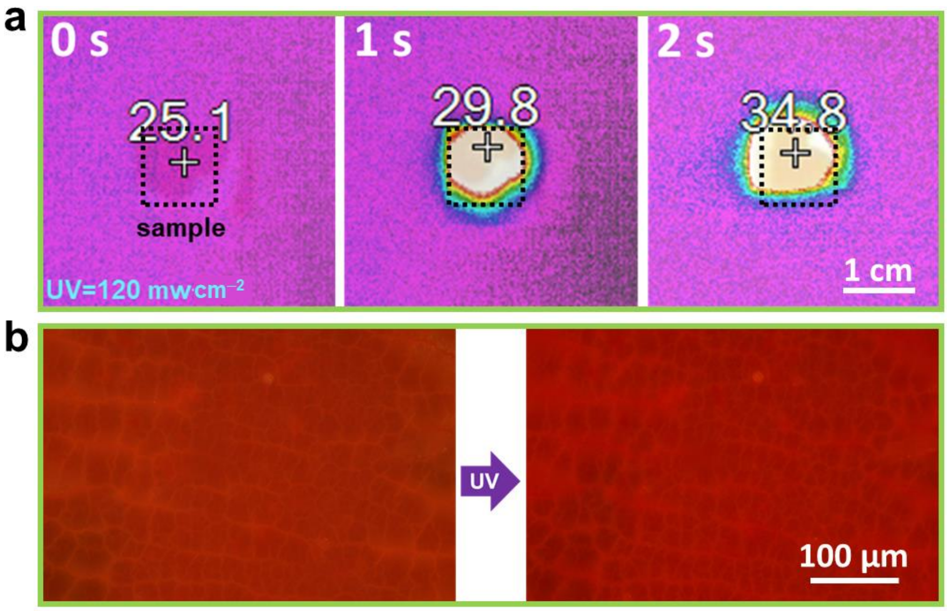

Figure S5. (a) The temperature change of AZOIOs under UV irradiation (120 mW/cm^2, 2 s). (b) POM images of AZOIOs before and after UV irradiation (120 mW/cm^2, 2 s).

2. The modulation effect of AZOIOs' bandgap and photo-isomerization on fluorescent property

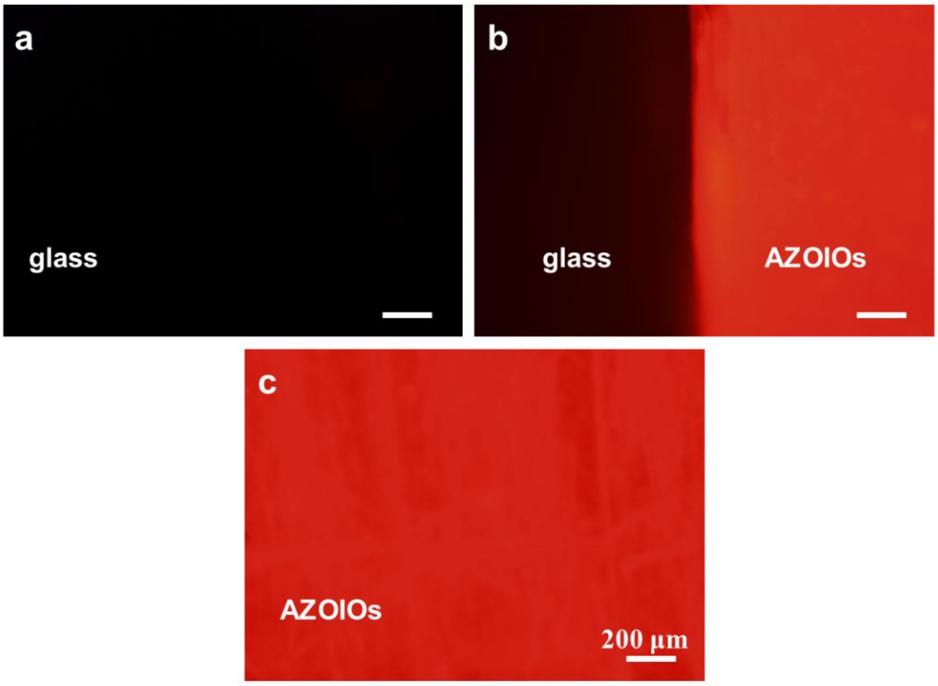

Figure S6. Fluorescent images of (a) glass, (b) the boundary of AZOIOs, and (c) AZOIOs.

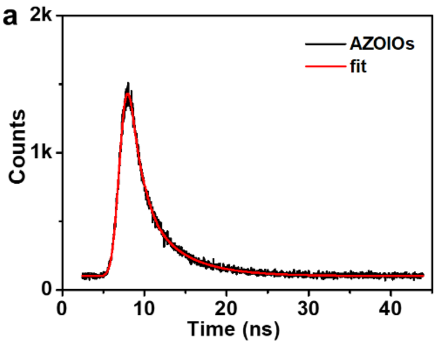

b

❖Exponential Components Analysis (Reconvolution)

Fitting range	[100; 1800] channels
χ^2	1.067

	B_i	ΔB_i	f_i (%)	Δf_i (%)	τ_i (ns)	$\Delta\tau_i$ (ns)
1	0.0241	0.0005	41.862	3.097	0.790	0.041
2	0.0059	0.0001	58.138	1.373	4.499	0.003

Shift	-0.035	(±	0.356	ns)
Decay Background	102.300	(±	0.425	)
IRF background	11.400			

Luminescence decay = 0.79×41.862%+4.499×58.138%=2.95 ns

Figure S7. The luminescence decay of AZOIOs when excited at 560 nm.

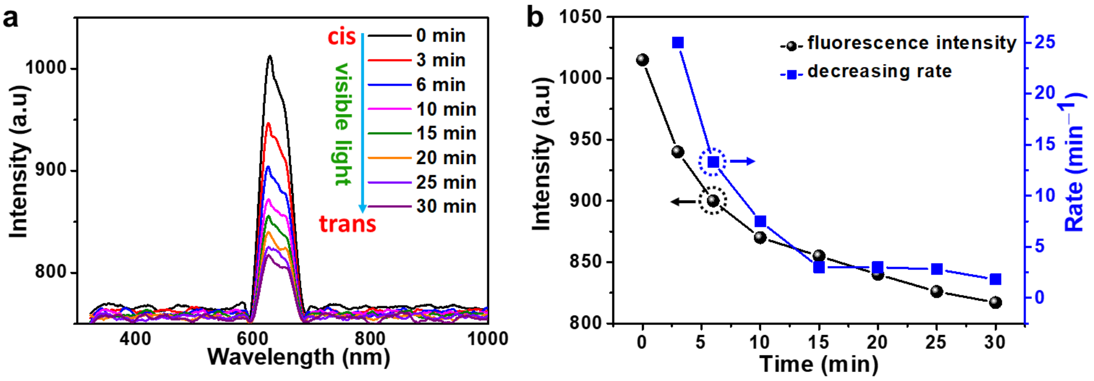

Figure S8. (a) The fluorescent intensity changes of the *cis*-bulk azobenzene polymer (a fresh bulk azobenzene polymer irradiated by 30 mW/cm^2 UV light for 1 min) under visible irradiation (10 mW/cm^2) for different time. (b) The transformation of fluorescent intensity and fluorescent decreasing rate for *cis*-bulk azobenzene polymer as a function of visible irradiation time.

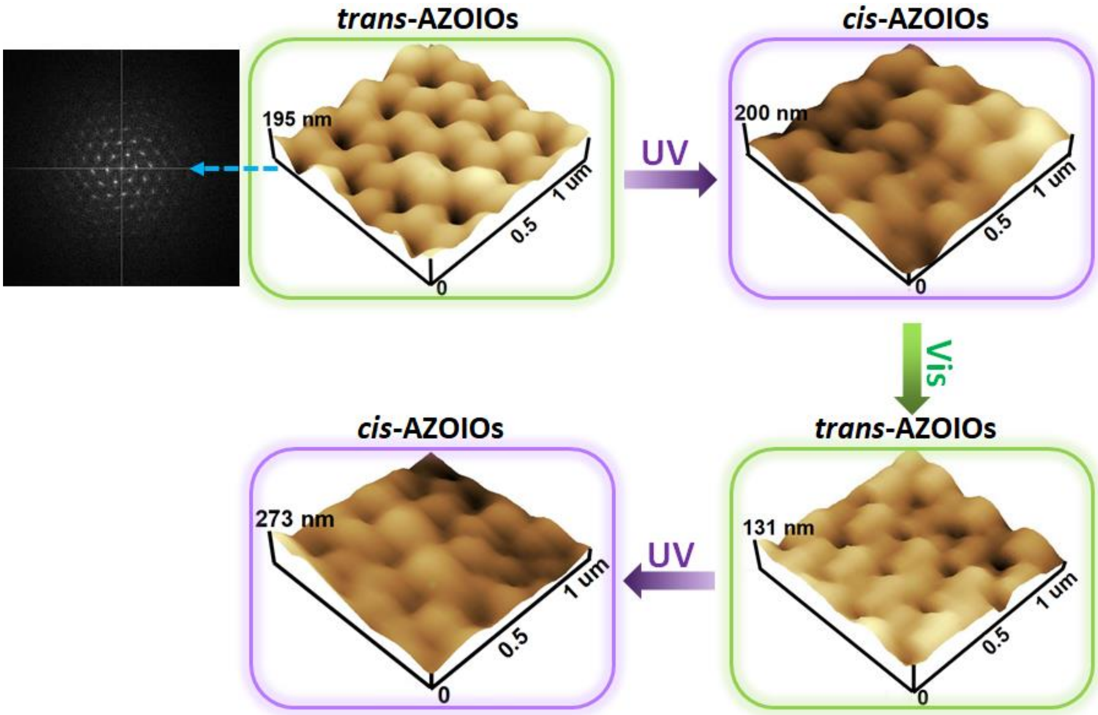

Figure S9. AFM images of the AZOIOs under alternating UV/vis irradiation and the corresponding Fourier transform.

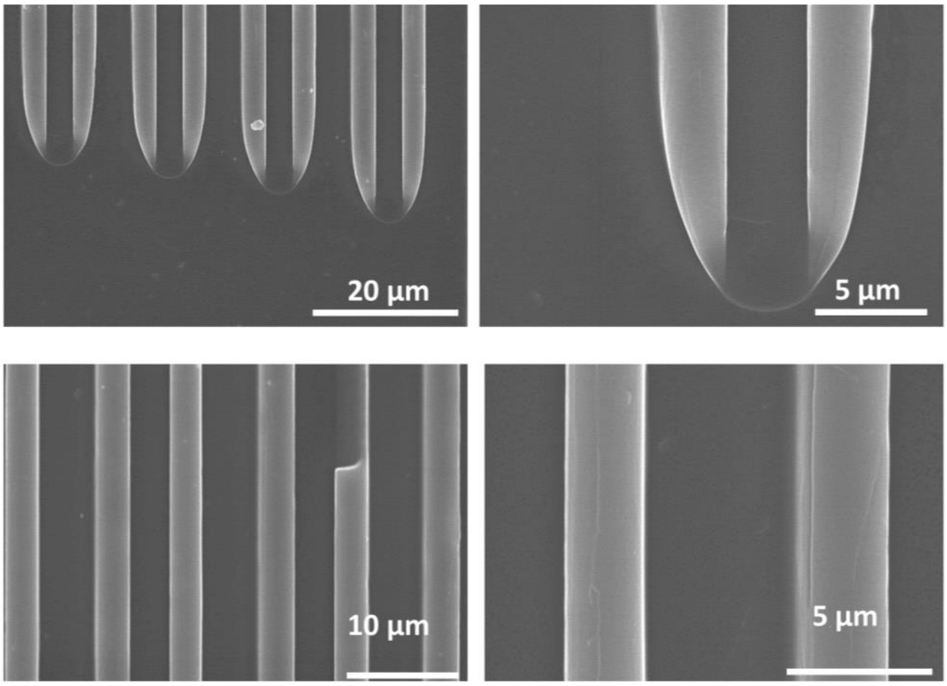

Figure S10. SEM images of the patterned bulk azobenzene polymer on a cover glass.

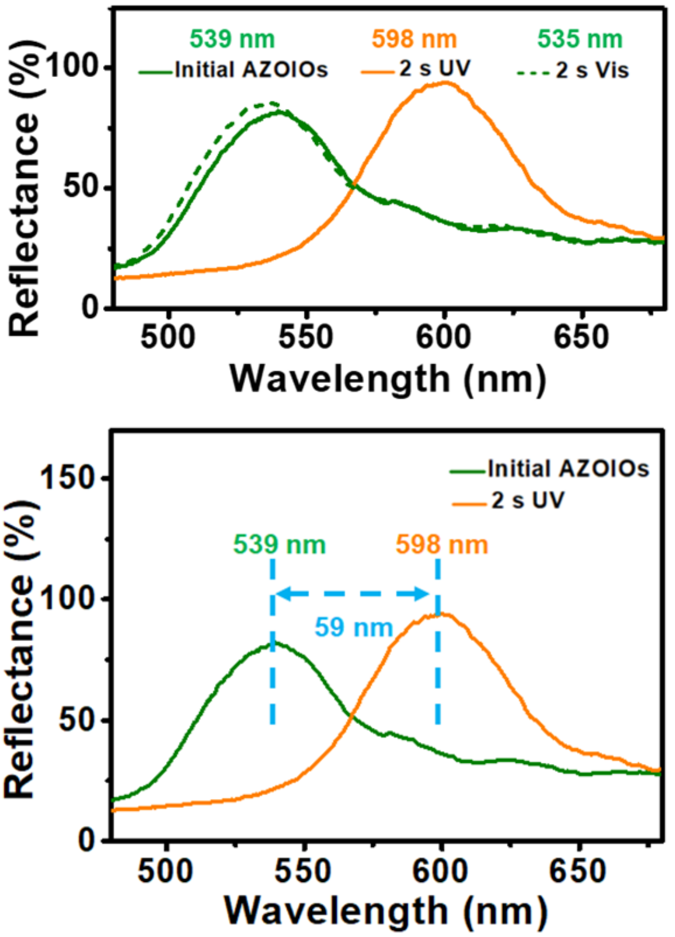

Figure S11. Reversible bandgap shift of AZOIOs under alternating UV (365 nm, 120 mW/cm^2) and visible (560 nm, 120 mW/cm^2) irradiation.

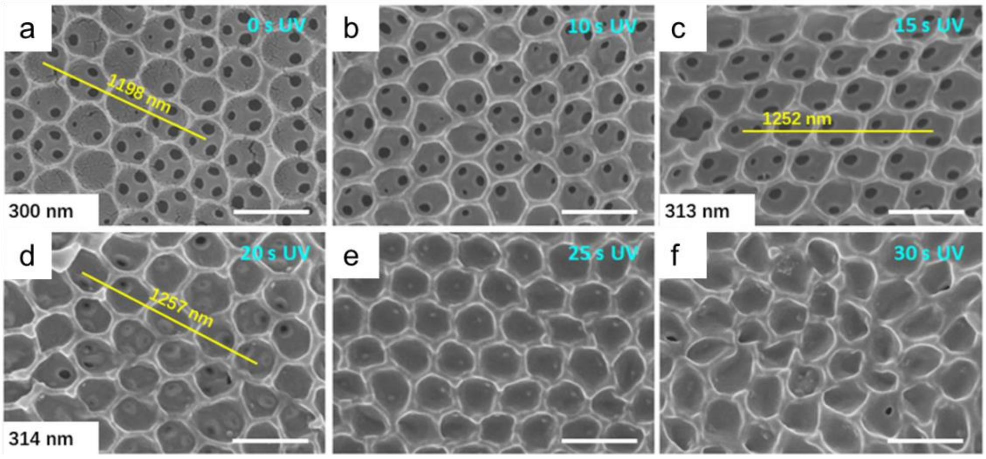

Figure S12. SEM images of the AZOIOs after UV irradiation (20 mW/cm^2) with different time. (a) 0 s; (b) 10 s; (c) 15 s; (d) 20 s; (e) 25 s; (f) 30 s.

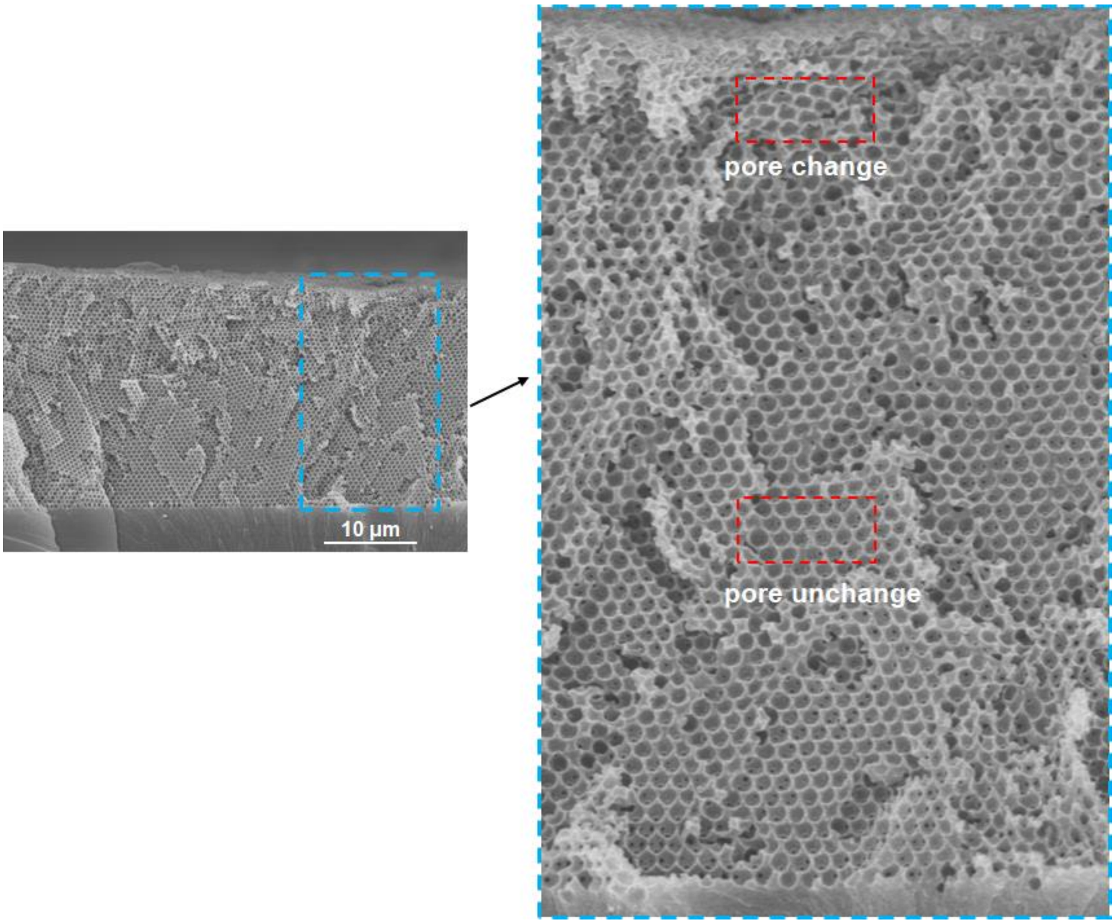

Figure S13. Side-view SEM images of the AZOIOs after UV irradiation (20 mW/cm^2, 20 s).

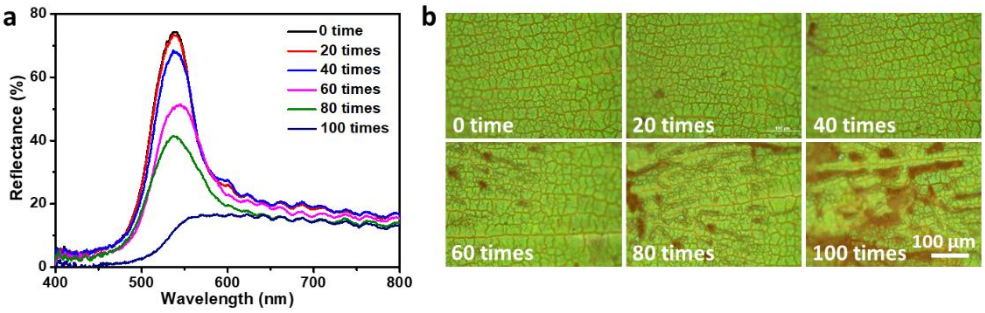

Figure S14. In-situ observation of (a) reflection spectra and (b) the corresponding optical images of AZOIOs after different abrasion times.
